# The germ layer: foundation of mesenchymal stem cell therapeutic destiny

**DOI:** 10.1038/s41419-026-09163-5

**Published:** 2026-08-07

**Authors:** Jiaxuan Wang, Xinyi Huang, Junli Zhou, Qianyi Shi, Yanjun Liu, Shoucheng Chen, Fang Huang, Yifan He

**Affiliations:** Hospital of Stomatology, Guanghua School of Stomatology, Sun Yat-sen University, South China Center of Craniofacial Stem Cell Research, Guangdong Provincial Key Laboratory of Stomatology, Guangzhou, China

**Keywords:** Mesenchymal stem cells, Stem cells

## Abstract

Stem cells represent a pivotal resource in clinical applications. Accumulating evidence indicates that their functional properties are closely associated with their tissue origin, particularly embryonic germ layer specification, which may govern the developmental heterogeneity and microenvironmental niche preferences of stem cells. This review provides a systematic comparison of stem cells originating from the surface ectoderm, neural crest ectoderm, and mesoderm, highlighting their distinct molecular signatures, functional properties, and therapeutic utility. These stem cells exhibit conserved expression of core pluripotency factors while simultaneously maintaining germ layer-specific markers that dictate their functional divergence in tissue regeneration, immunomodulation, apoptosis regulation, and angiogenic modulation. Stem cells acquire germ layer-specific regulatory characteristics that intrinsically determine their biological destiny, including their biological functions, differentiation potential, and clinical applicability. This review provides a deeper understanding of germ layer-dependent properties, which will enhance the rational selection of stem cell sources for targeted therapies.

## Highlights

- Based on existing literature, this article reviews stem cells originating from the surface ectoderm, neural crest ectoderm, and mesoderm. A critical priority is to systematically profile stem cells from various germ layers and conduct comparative studies to validate a germ layer-based strategy for clinical stem cell selection.
- To better elucidate the fundamental properties and therapeutic relevance of stem cells, this article proposes a transition shift from a tissue-of-origin to a germ layer-based classification. Within this novel framework, we systematically review the surface markers, biological functions, and clinical applications of various stem cells.
- While stem cell therapy shows enhanced efficacy in tissues derived from the same germ layer—likely reflecting conserved genetic signatures—the biological characteristics of distinct stem cell types also substantially modulate their therapeutic potential.

## Introduction

Stem cells are characterized as a group of cells that possess the potential for differentiating into multiple cell lineages and the ability to self-renew. Their functional identities emerge during the pivotal event of gastrulation, wherein morphogenetic gradients partition the embryo into ectodermal, mesodermal, and endodermal territories. This spatial and molecular segregation installs enduring epigenetic blueprints that govern stem cell behavior throughout life. Consequently, adult stem cells retain an indelible developmental memory that dictates their differentiation biases, microenvironmental dependencies, and reparative functions—attributes profoundly overlooked in conventional tissue-centric classification systems.

Mesenchymal stem cells (MSCs) are widely distributed in the body and play crucial roles in tissue regeneration and homeostasis. They can be isolated from discarded tissues, expanded in vitro, and utilized as therapeutics for autoimmune diseases and chronic disorders by modulating immune cell activity [1]. There has been increasing awareness that the tissue origin of stem cells is intrinsically linked to their functions [2–4]. Generally, stem cells harvested from a specific organ are capable of repairing damage to the same target organ [5, 6]. These cells exhibit intrinsic properties reflective of their derived tissue. For instance, bone marrow mesenchymal stem cells (BMMSCs) are renowned for their robust osteogenic differentiation potential [7], while neural stem cells are celebrated for their exceptional ability to differentiate into neural lineages [8]. Consequently, in order to enhance the efficacy of stem cell-based therapies, researchers frequently endeavour to utilize stem cells of identical tissue or organ provenance for treatment [9–11]. However, the exclusive reliance on tissue origin to determine the clinical application of stem cells presents several limitations. These include the challenges in obtaining stem cells from specific tissues, such as the liver and kidney, as well as the ethical concerns surrounding the procurement of these stem cells from human subjects [12]. Consequently, the selection of alternative stem cell sources for treatment and the achievement of satisfactory therapeutic outcomes remain significant challenges in the field of stem cell applications.

Besides classifying stem cells according to their organ or tissue source, the germ layer origin of stem cells can also serve as a criterion for classification. Stem cells have a wide variety of germ layer origins. For instance, BMMSCs, adipose mesenchymal stem cells (AMSCs), and umbilical cord mesenchymal stem cells (UCMSCs) originate from the mesoderm [13–17]. Stem cells originating from the neural crest ectoderm include stem cells from human exfoliated deciduous teeth (SHEDs), periodontal ligament stem cells (PDLSCs), and dental pulp stem cells (DPSCs) [18–23]. Additionally, limbal epithelial stem cells (LESCs) originate from surface ectoderm [24, 25]. In rat and rabbit calvarial bone (derived from neural crest ectoderm) defect models, stem cells from the same germ layer have demonstrated higher new bone formation ability than BMMSCs originating from the mesoderm [13, 26]. In addition, dental mesenchymal stem cells (DMSCs) originating from neural crest ectoderm also possess enhanced therapeutic efficacy when regenerating tissues from the same germ layer, such as nerve and periodontal tissue, compared to stem cells originating from other germ layers [27–31]. These studies suggest that the germ layer origin of stem cells could be a more advantageous predictor of cellular functionality. Consequently, the categorization of stem cells according to their germ layer origin might emerge as a promising criterion for directing their clinical utilization. This review presents a summary of the germ layer origins and functions of diverse stem cells. The objective is to offer additional information and establish a research basis for the clinical applications of stem cell therapy in disease treatment.

## Biological characteristics of stem cells from different germ layers

**Fig. 1:**
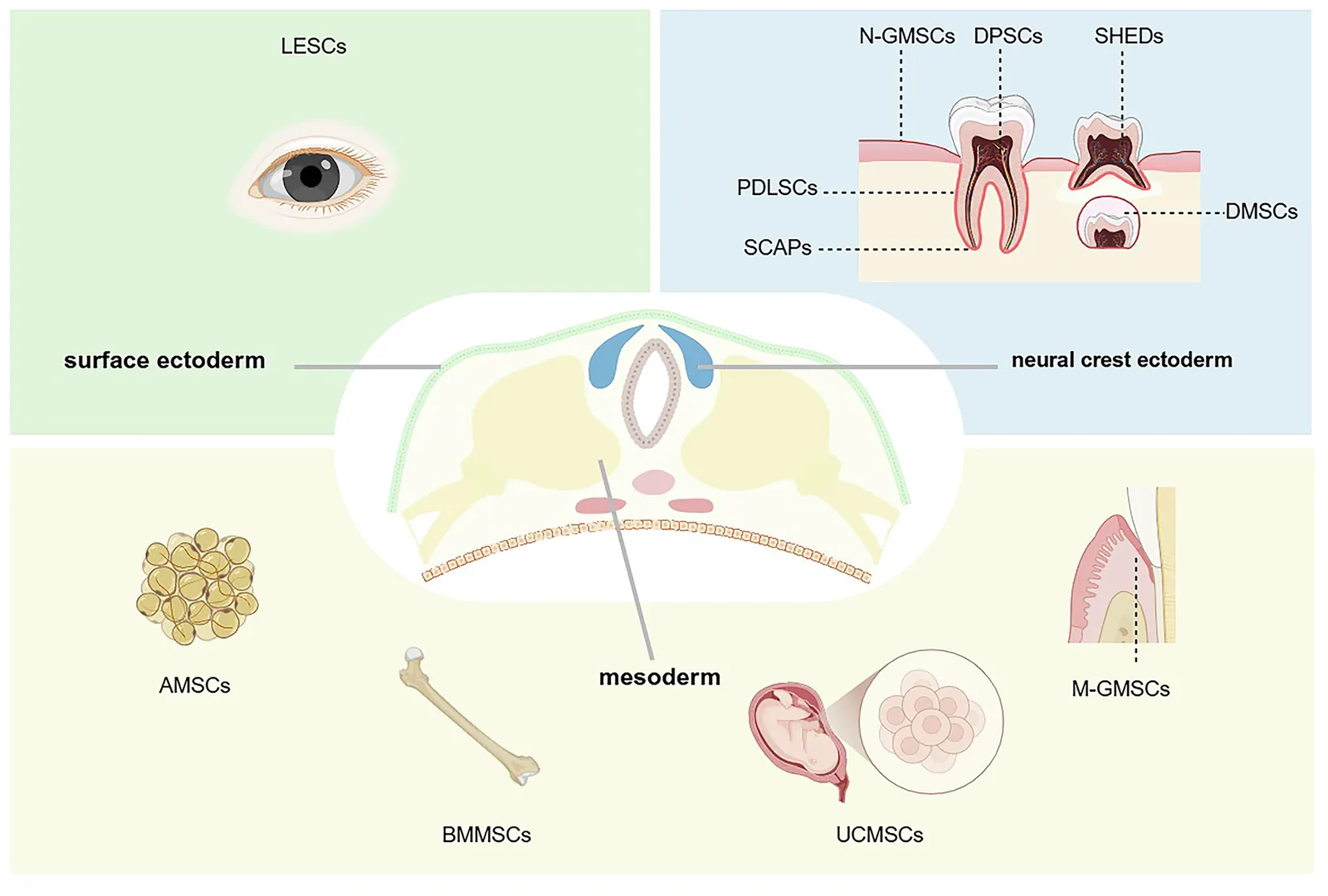
Stem cells originating from different germ layers. Within the framework of developmental biology, stem cells can be classified based on their embryonic layer origin. In this article, the classification primarily includes stem cells originating from the surface ectoderm, neural crest ectoderm, and mesoderm. The green area represents stem cells originating from the surface ectoderm, including LESCs. The blue area represents stem cells from the neural crest ectoderm, including SHEDs, DPSCs, PDLSCs, DFSCs, SCAPs, and N-GMSCs. The yellow area represents stem cells originating from the mesoderm, including BMMSCs, AMSCs, UCMSCs, and M-GMSCs.

Traditionally, embryonic tissues are regarded as originating from the three primary germ layers: the ectoderm, the mesoderm, and the endoderm. Although the neural crest originates from the ectoderm, it is increasingly recognized as the ‘fourth germ layer’ due to its pluripotency and significant contributions to development [32], with the ability to differentiate into various stem cell types such as DMSCs [33]. To make a distinction, the ectoderm is classified into surface ectoderm and neural crest ectoderm in this review. However, the precise germ layer origin of many stem cells remains unclear, particularly those derived from the endodermal tissues. For example, stem cells have been identified in endodermal organs such as the liver, lung, and pancreas, but research into their precise germ layer origin remains limited due to technical challenges in culturing these cells derived from human tissues in vitro, as well as ethical concerns [12]. Therefore, here we delineate stem cell populations based on their provenance from the three germ layers: the surface ectoderm, the neural crest ectoderm, and the mesoderm, and subsequently elucidate their biological characteristics (Fig. 1,Table 1). In particularly, surface markers serve as tools for characterizing cellular functions, with precise identification typically requiring a combination of multiple markers. Consequently, identifying a distinct stem cell population based solely on a single marker remains challenging due to their inherently heterogeneous and dynamic nature [34].

**Table 1:** Germ layer, distribution location, and surface markers of stem cells.

Germ layer	Stem cells	Tissue	Surface markers	References
Surface ectoderm	LESCs	The limbal region of the corneal epithelium	CD71, ΔNp63, KRT15, α6 integrins, ABCG2, Bmi1, and N-cadherin	[24, 25]
Neural crest ectoderm	SHEDs	Exfoliated deciduous teeth	CD73, CD90, CD105, STRO-1, CD44, CD146, CD10, OCT4, NANOG, nestin, ncam1 and β3 tubulin	[51, 52]
	DPSCs	Dental pulp tissues of human extracted permanent teeth	CD73, CD90, CD105, STRO-1, CD44, CD10, OCT4, NANOG, nestin, ncam1, β3 tubulin and PD-1	[51, 52, 65]
	PDLSCs	Periodontal ligament	CD73, CD90, CD105, CD29, CD44, STRO-1, CD146, CD10, CD26, CD166, OCT-4, NANOG, SSEA-1, SSEA-4 and nestin	[54]
	SCAPs	Apical papilla	CD73, CD105, CD90, STRO-1, CD146, CD29, CD166, CD106 CD24 and PD-1	[22, 56, 66]
	DFSCs	Dental follicle	CD73, CD90, CD105, Notch-1, CD44, STRO-1, OCT4, NANOG, nestin, β3 tubulin, p75 and HNK1	[57]
	N-GMSCs	Gingiva	CD44, CD90, CD105, CD73, and Sca-1	[50, 58, 59]
Mesoderm	BMMSCs	Bone marrow	CD73, CD90, CD105, CD13, CD44, CD106, CD146, CD166, Sca-1, SSEA-4, CD49a, STRO-1 and CD271	[74–76, 279, 280]
	AMSCs	Adipose	CD73, CD90, CD105, CD13, CD29, CD44, CD146, CD166, CD36, CD10 and CD200	[77, 78, 85]
	UCMSCs	Umbilical cord including cord lining, perivascular tissue, and Wharton’s jelly	CD73, CD90, CD105, CD13, CD29, CD44, CD106, CD146, CD166 and EphA2	[79–82, 89]
	M-GMSCs	Gingiva	CD44, CD90, CD105, CD73, and Sca-1	[50, 58, 59]

Comparative profile of representative stem cell types originating from three embryonic germ layers (surface ectoderm, neural crest ectoderm, and mesoderm). Listed are specific tissue niches, primary surface markers, and key literature references.

### Stem cells originating from the surface ectoderm

The surface ectoderm consists of a continuous and thick layer of epidermal progenitors, which start to emerge around the gastrulation stage. Eventually, they develop into the epithelia of the eye, nasal cavity, mouth, and skin, along with their appendages [35]. These appendages include hair, mammary glands, sweat glands, and salivary glands [36]. It is noteworthy that a proportion of these cells retain stem cell characteristics, such as LESCs, which are located in the limbal region of the corneal epithelium [24, 25].

LESCs express stem cell surface marker CD71, and surface ectoderm-associated markers including P63, keratin 15, and α6 integrin [24, 25]. These ectodermal markers are also expressed in the surface ectodermal progenitor cells [37], where they are implicated in critical biological processes, including the proliferation and differentiation of epithelial cells, the maintenance and migration of stem cells, as well as the formation of tissues [38–40]. Notably, stage-specific embryonic antigen 4 (SSEA4) is a marker of pluripotent human embryonic stem cells and serves as a negative marker for LESCs [41, 42]. Therefore, the absence of SSEA4 indicates that LESCs may lack the potential to differentiate into somatic cells of other germ layers. Furthermore, LESCs exhibit additional specific markers including ABCG2, Bmi1, and N-cadherin, which serve a function of transporting endogenous molecules and xenobiotics [43], maintaining redox homeostasis and self-renewal [44], and mediating cell adhesion [45].

### Stem cells originating from the neural crest ectoderm

Neural crest cells (NCCs) arise from the dorsal region of the fusing neural tube and subsequently undergo delamination from the neuroepithelium. These cells then migrate over substantial distances in distinct streams, with migration patterns characteristic of their position along the anterior-posterior axis of the embryo [46]. The initial phase of NCCs migration is observed in broad streams, beginning in the cephalic region. Following this, trunk migration is initiated in narrower streams along the anterior-posterior axis, proceeding in a progressive manner and forming a chain-like structure [47]. Subsequent to migration, NCCs undergo a process of differentiation, resulting in the formation of a diverse array of cell types. These cells then contribute to the development of various organs, with this process occurring in a region-specific manner [48]. For instance, cephalic NCCs travel long distances within the embryo and precisely migrate to specific branch arches, where they contribute to the formation of innervations, bones, cartilage, connective tissues, and muscles, ultimately forming various tissue structures in the craniomaxillofacial region, including dentin pulp, cementum, periodontal ligaments, and the mandible [47, 49].

DMSCs, comprising SHEDs, DPSCs, PDLSCs, dental follicle stem cells (DFSCs), and stem cells from the apical papilla (SCAPs), as well as 90% of gingival mesenchymal stem cells (GMSCs), developmentally originate from the neural crest ectoderm [18–23, 50]. These cells express mesenchymal surface markers (CD73, CD90, CD105) while lacking hematopoietic markers (CD34, CD45) [22, 50–59]. Due to their embryological neural crest origin, DMSCs have been observed to exhibit expression of both neural crest-related markers (β3-tubulin, nestin) and pluripotent markers (Oct4, NANOG) [52, 54, 57, 58]. This embryonic origination grants DMSCs enhanced neuroectodermal differentiation potential (e.g., Schwann cells, neurons) alongside tri-lineage multipotency, enabling in vitro differentiation into endodermal (e.g., insulin-producing cells) and mesodermal lineages (e.g., osteocytes, adipocytes, chondrocytes) [58, 60].

Despite their shared origin in the germ layer, these cells are distributed in different locations within the tooth and oral mucosa, and their characteristics and properties vary accordingly. It is noteworthy that both SHEDs and DPSCs are derived from the dental pulp; however, there is a distinction in their origin. SHEDs are sourced from deciduous teeth, while DPSCs are derived from permanent teeth. Consequently, SHEDs show elevated expression levels of some pluripotent markers, including NANOG and OCT4, whereas DPSCs demonstrate increased expression of neuroectodermal markers, such as PAX6 and nestin [61]. Wang et al. [62] also found that SHEDs showed an enhanced proliferation rate and differentiation potential compared to DPSCs in vitro. Moreover, in vivo transplantation outcomes indicated that SHEDs had an enhanced mineralization ability relative to DPSCs [62]. These findings suggest that SHEDs are relatively undifferentiated stem cells. Furthermore, nestin gene expression is markedly elevated in DPSCs compared to PDLSCs, which may indicate a propensity for DPSCs to differentiate into odontogenic cells, considering that nestin functions as a marker for all DPSCs and odontoblasts [63]. It is also notable that CD24 is a specific surface marker of SCAPs, since it has been found undetectable in other MSCs [56]. During in vitro odontogenic differentiation, SCAPs have been observed to lose CD24 expression while increasing the expression of odontogenic differentiation markers including ALP [56]. Consequently, CD24 can be used as a marker of undifferentiation, and its presence is associated with enhanced stemness of SCAPs [64]. Intriguingly, programmed cell death protein-1 (PD-1) is a transmembrane protein that plays a crucial role in immunoregulation. Recent studies have indicated that PD-1, expressed on DPSCs and SCAPs, can activate the SHP2 signal to maintain proliferation and inhibit osteo/odontogenic differentiation [65, 66]. In contrast to DPSCs, DFSCs exhibit reduced differentiation propensity, correlating with elevated expression of pluripotency regulators (OCT4, SOX2) and immunomodulatory mediators (IL-6, TLR4) [67]. Lima et al. further characterized DFSCs as displaying moderate expression of neuroectodermal markers p75 and HNK1, suggesting retention of undifferentiated neural crest cells in the dental follicle during odontogenesis [57]. This observation supports that the low differentiation of DFSCs may be associated with the cellular origin of undifferentiated neural crest cells. CD146, a transmembrane glycoprotein expressed on MSCs (DPSCs, SCAPs, SHEDs) and vascular cells (endothelial cells, pericytes), serves as a critical regulator of differentiation plasticity and angiogenic competence [68–71]. Comparative analyses reveal heightened CD146 expression in SHEDs versus DPSCs, paralleling the enhanced angiogenetic capacity of SHEDs [70]. Compared to GMSCs originating from the mesoderm (M-GMSCs), GMSCs originating from neural crest ectoderm (N-GMSCs) demonstrate superior neural/chondrogenic differentiation and activated T-cell apoptosis induction in vitro. This immunomodulatory superiority associates with FAS ligand (FASL) upregulation in N-GMSCs [50]. Furthermore, the neuroectodermal origin of N-GMSCs confers unique multipotency, distinct migratory, and contractile phenotype, mechanistically linked to scarless mucosal healing—a distinctive feature of oral wound repair [72].

### Stem cells originating from the mesoderm

The mesoderm, situated between the ectoderm and endoderm during gastrulation, is one of the three primary germ layers. This lineage differentiates into core body systems, including the skeletal, muscular, excretory, circulatory, lymphatic, and reproductive systems, as well as connective tissues, the dermis of the skin, body cavity linings, the adrenal cortex, and various internal organs [73]. Mesodermal progenitors further generate multiple specialized cell populations, including BMMSCs [13, 14], AMSCs [15], UCMSCs [16], and a subset (10%) of GMSCs [50]. These stem cells, originating from the mesoderm, consistently express consensus mesenchymal markers (CD73, CD90, CD105, CD44, CD13, CD29, CD166, CD106, CD146) [74–82] while lacking hematopoietic lineage markers (CD34, CD45, HLA-DR) [83].

Crucially, homogeneity of the germ layer does not preclude cellular heterogeneity. Although sharing mesodermal origin, distinct stem cell populations exhibit divergent surface marker profiles and functional attributes. For instance, Camilleri et al. identified nine non-canonical markers (CD200, CD163, CD36, CD274, CD271, CD146, CD248, CD276, CD140b) which may be used to further characterize AMSCs [84]. Among these markers, CD10 and CD200 have been identified as potential specific markers of AMSCs, which are depot-dependent and associated with adipogenic capacities [85]. Furthermore, AMSCs possess enhanced proliferative capacity and higher immunomodulatory effects under human platelet lysate-supplemented culture conditions compared with BMMSCs, while BMMSCs exhibit greater potential for osteogenic and chondrogenic differentiation [86]. Similarly, a lower expression level of human leukocyte antigen class I antigen is observed in AMSCs in comparison with BMMSCs [86]. Additionally, studies have demonstrated that UCMSCs possess anti-cancer capabilities [87, 88]. In recent years, Al Madhoun et al. have reported that Ephrin receptors A2 (EphA2) function as a specific cell surface marker for UCMSCs [89]. Ephrin receptors and their ligands are vital for embryonic development; however, they are also overexpressed in numerous malignant neoplasms, where they promote carcinogenesis [90]. The expression of EphA2 on UCMSCs may be linked to their anti-cancer potential, although the exact nature of this relationship has yet to be clarified.

## The biological functions of stem cells originating from different germ layers

Since the biological characteristics of stem cells have been elucidated, it is reasonable to inquire how cells contribute to the treatment of various diseases during the pathogenesis process. In the aftermath of transplantation, stem cells and their secretome, including extracellular vesicles (EVs), apoptotic vesicles (apoVs), and soluble factors, play a pivotal role in the process of tissue repair. Below, we clarify the biological functions of stem cells through tissue regeneration, immunoregulatory effects, apoptosis modulation effects, and angiogenic modulation effects.

### Tissue regeneration

Numerous studies have demonstrated the pivotal role of stem cell therapy in tissue repair, such as in skin wounds, myocardial infarction, and renal injury [91–93]. These cells predominantly facilitate tissue regeneration through two mechanisms: by differentiating into somatic cells and by secreting growth factors and EVs.

In tissue repair processes, stem cells play a critical role in replenishing lost cells *via* proliferation and differentiation [94]. Originating from the surface ectoderm, LESCs can generate corneal epithelial cells, thereby rejuvenating and maintaining the corneal surface after simple limbal epithelial transplantation [95]. Furthermore, Wright et al. demonstrated that LESCs encapsulated in alginate secreted galectin-1 and secreted protein acidic and rich in cysteine (sPARC), which were capable of regulating corneal epithelial cell proliferation [96].

Stem cells originating from the neural crest ectoderm also have the potential to undergo differentiation, with their fate modulated by microenvironmental soluble factors [97]. Key regulatory factors include epidermal growth factor (EGF), nerve growth factor (NGF), and insulin-like growth factor-1 (IGF-1) [21, 23, 98, 99]. For example, IGF-1 has been demonstrated to promote the osteogenic differentiation and bone formation of PDLSCs through ERK/JNK MAPK signaling pathways [100]. Li et al. identified that a combination of NGF, basic fibroblast growth factor (bFGF), L-glutamine, IGF-1, and EGF could induce the differentiation of PDLSCs into neuronal lineage [99]. Stem cells can also contribute to tissue repair by secreting growth factors and EVs. DPSCs and SCAPs secrete neurotrophic factors such as NGF, glial cell line-derived neurotrophic factor (GDNF), and brain-derived neurotrophic factor (BDNF), which facilitate recovery from nerve injury or degeneration [101, 102]. DMSC-conditioned medium contains bone morphogenetic protein 7 and dentin sialophosphoprotein, alongside regulatory proteins such as multiple inositol polyphosphate phosphatase 1, WNT1-inducible-signaling pathway protein 2, and enamelin, which collectively orchestrate bone formation, turnover, endochondral ossification, and mineralization [103]. Swanson et al. also provided evidence that DPSC-EVs enhance the differentiation and mineralization of BMMSCs, thereby exerting osteogenic capabilities [104].

**Fig. 2:**
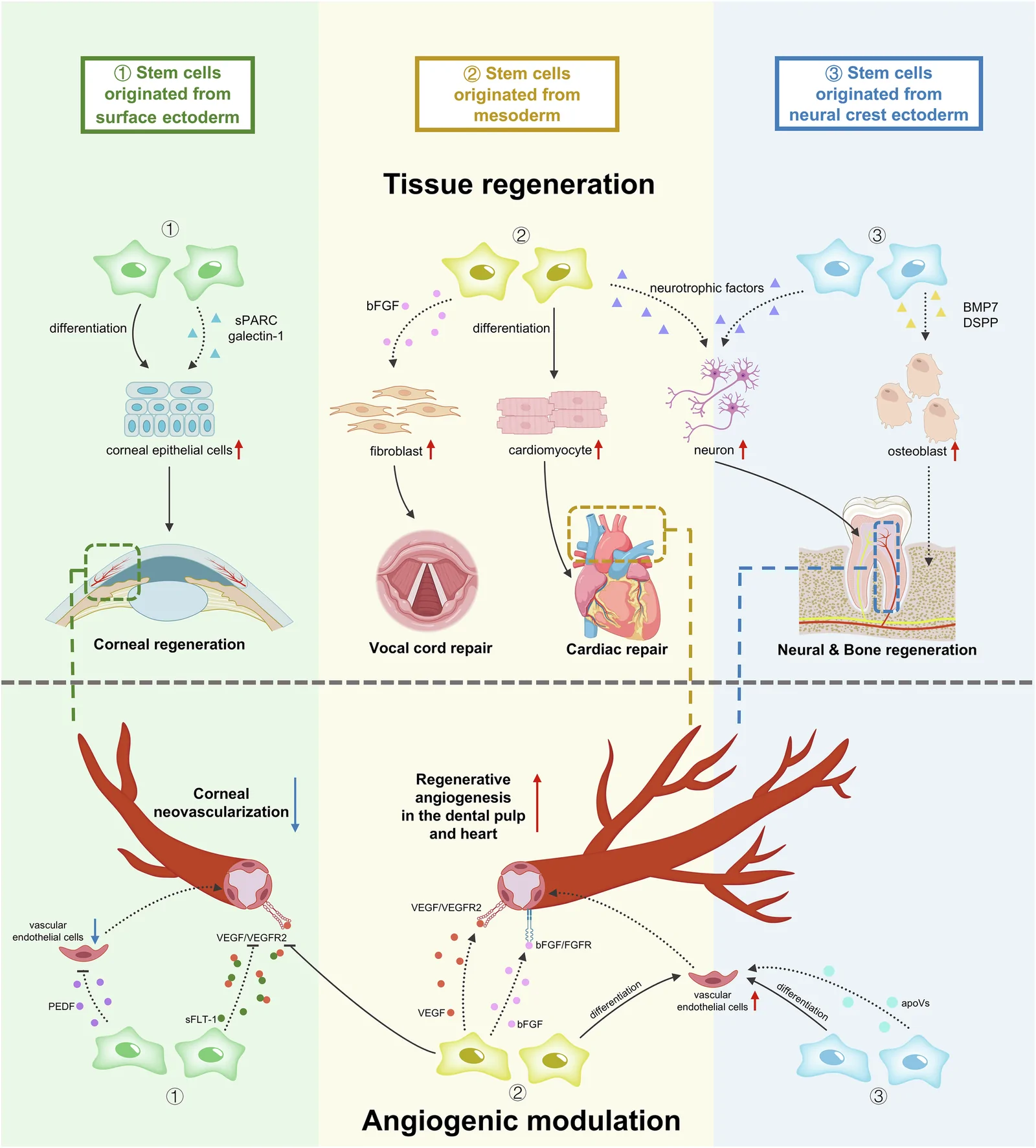
Tissue regeneration and angiogenic modulation mediated by stem cells originating from distinct embryonic lineages. Stem cells originating from the surface ectoderm, neural crest ectoderm, and mesoderm participate in tissue repair through differentiation into lineage-specific cells and through the secretion of growth factors and EVs. LESCs originating from the surface ectoderm contribute to corneal epithelial maintenance and regeneration. Stem cells originating from the neural crest ectoderm (e.g., DPSCs, SCAPs, SHEDs) undergo fate specification in response to microenvironmental cues and secrete bioactive factors that support neural repair, mineralized tissue formation, and tissue regeneration. Stem cells originating from the mesoderm (e.g., BMMSCs, AMSCs, UCMSCs) contribute to tissue repair through direct differentiation and secretome-mediated effects in injured tissues.

For stem cells originating from the mesoderm, the secretome including growth factors, cytokines, and EVs, possesses the capacity to rescue injured cells, reduce tissue damage, and accelerate tissue repair [105]. BMMSCs can alleviate deafferentation pain in rats through secreting GDNF, which promotes the activation of the PI3K/AKT signaling pathway [106]. In mice with myocardial infarction, genetically modified BMMSCs reduce pathological remodeling and improve cardiac function by secreting frizzled-related protein (sFRP), which functions as an inhibitor of the Wnt signal pathway and is capable of decreasing cardiomyocyte apoptosis [107]. Moreover, AMSCs can secrete hepatocyte growth factor (HGF) and bFGF, thus promoting the proliferation of injured vocal fold fibroblasts in vocal fold injury patients [108]. The secretome of AMSCs, such as neurotrophin (NT)-4, ciliary neurotrophic factor (CNTF), bFGF, HGF, and IGF-1 [109], also exerts pivotal influences on the formation of the nervous system such as synapse formation and axonal guidance (Fig. 2) [110].

### The immunoregulatory effects of stem cells

The immunomodulatory mechanisms of stem cells are mediated through direct cell-to-cell contact, soluble factors, and EVs in response to immune cells [1]. Rather than ranking the relative performance of various MSC types, this discussion highlights that stem cells from diverse tissue origins universally possess these regulatory capacities, albeit operating through distinct underlying mechanisms.

Stem cells originating from the neural crest ectoderm exert immunomodulatory effects *via* direct cell-to-cell contact. In Sjögren’s syndrome, transplanted SHEDs induce T helper 17 cells (Th17) apoptosis *via* direct cell-cell contact, thereby modulating the Regulatory T cells (Treg)/Th17 balance [4]. Similarly, PDLSCs are capable of suppressing B cell activation mediated through the interaction of PD-1 and PD-L1 [111]. Additionally, soluble factors also play a crucial role in immunoregulation. In neural diseases, transplanted PDLSCs secrete anti-inflammatory factors interleukin (IL)-10 and transforming growth factor-beta (TGF-β) [98], while SHEDs and DPSCs upregulate the secretion of anti-inflammatory factors such as TGF-β, IL-6, IL-4, IL-1, IL-8, and indoleamine 2,3-dioxygenase (IDO) [101]. In vitro, DPSCs and PDLSCs inhibit activated CD4 + /CD8 + T cell proliferation through a mechanism dependent on IFN-γ/TNF-α signaling, MSC PD-L1 upregulation, and elevated IL-10 levels [112]. GMSCs have been demonstrated to polarize macrophages toward the M2 phenotype [113]. Furthermore, microRNAs encapsulated within EVs serve as key regulators of immune responses. Studies reported that DPSCs secreted miR-1246, which inhibited Nfat5 to modulate the polarization of activated Th17 [114, 115], contributing to calvarial bone regeneration [20]. Zheng et al. also demonstrated that PDLSCs inhibited Th17 *via* the miR-155-5p/SIRT1 signaling pathway, thereby promoting periodontal tissue regeneration [116].

**Fig. 3:**
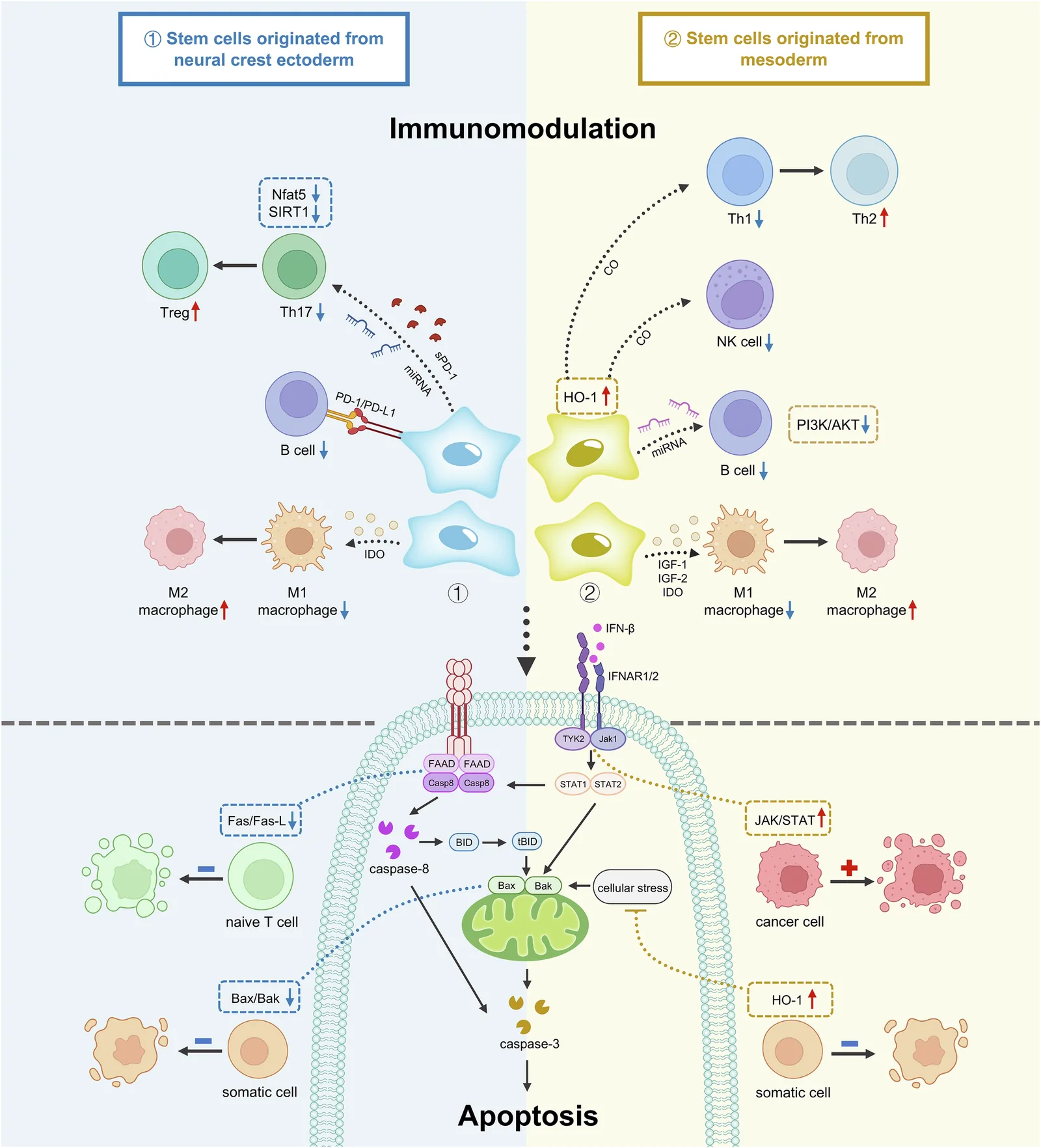
Immunomodulatory and apoptosis-regulatory effects of stem cells originating from distinct embryonic lineages. Stem cells originating from the neural crest ectoderm and mesoderm exert immunomodulatory effects through direct cell-to-cell contact, soluble factors, and EVs in response to immune cells. Stem cells originating from the neural crest ectoderm (e.g., SHEDs, DPSCs, PDLSCs, GMSCs) regulate immune homeostasis by modulating T-cell subsets, suppressing B-cell activation, and promoting macrophage polarization toward anti-inflammatory phenotypes through mechanisms involving immune checkpoint signaling, immunoregulatory cytokines, metabolic enzymes, and EV-delivered microRNAs. Stem cells originating from the mesoderm (e.g., BMMSCs, AMSCs, UCMSCs) similarly regulate immune responses by inducing immune cell apoptosis, suppressing pro-inflammatory immune activity, and reshaping immune cell balance through contact-dependent and secretome-mediated pathways.

For stem cells from the mesoderm, BMMSCs mitigate inflammatory tissue injury by triggering T-cell apoptosis through the contact-dependent FasL/Fas pathway [117]. Furthermore, inflammatory immune regulation is also driven by soluble factors and EV-mediated delivery of immunoregulatory microRNAs and proteins [118]. For instance, BMMSCs reprogram macrophages toward the M2 phenotype through the upregulation of IGF-1, thereby decreasing neuronal apoptosis in global ischemia [119]. The overexpression of heme oxygenase-1 by BMMSCs has been shown to suppress natural killer cells, decrease the Th1/Th2 balance, and facilitate the shift of pro-inflammatory Th17 to immunosuppressive Tregs in vitro [120]. Moreover, BMMSC-EVs inhibit B cell proliferation and activation by downregulating the PI3K/AKT signaling pathway through the delivery of miR-155-5p [121]. Chen et al. further revealed that BMMSC-EVs treatment of peripheral blood mononuclear cells induces a phenotypic switch in helper T cells from a pro-inflammatory Th1 to a predominance of anti-inflammatory Th2 cells [122]. AMSCs can alleviate periodontitis in rat models through stimulating M2 macrophage polarization *via* the IDO-dependent Kyn-AhR-NRF2 pathway [123]. UCMSC-EVs have been demonstrated to diminish the synthesis of CCL2 and CCL21, leading to a notable decrease in CD4-expressing T cells and M2 macrophages within the injured retinae [124]. UCMSCs are capable of producing IGF-2, which preconditions macrophages to experience anti-inflammatory polarization [125]. Generally, it can be surmised that stem cells exert control over immune function through a multitude of mechanisms (Fig. 3).

### The apoptosis modulation effects of stem cells

Apoptosis is a regulated form of cell death that eliminates abnormal cells [126]. This process is controlled by various genes, including members of the B-cell lymphoma (Bcl)-2 and caspase protein families, as well as tumor suppressor genes and oncogenes [127]. During the pathological progression of certain conditions, including autoimmune and neurodegenerative diseases, abnormal apoptosis may lead to tissue damage [128].

Notably, stem cells can mediate therapeutic benefits by modulating the apoptosis process. For stem cells from the neural crest ectoderm, GMSC-conditioned medium protects injured cells from death by upregulating anti-apoptotic Bcl-2 and inhibiting pro-apoptotic Bcl-2 associated X (Bax) and cleaved caspase-3 [129]. DPSCs maintain Bcl-xL expression and suppress TNF-α overproduction, thereby disrupting both the intrinsic and extrinsic apoptotic pathways [101]. DPSC-EVs also inhibit apoptosis in human neuroblastoma cells by upregulating Bcl-2 and suppressing the pro-apoptotic protein Bax [130]. The mRNA encapsulated within EVs exhibits the ability to suppress apoptosis through the post-transcriptional regulation of target genes [131]. Beyond somatic cytoprotection, stem cells also regulate immune cell apoptosis. For instance, in atopic dermatitis, DFSCs suppress Fas and FasL expression in naive T cells, thereby attenuating inflammation [132].

In the context of MSCs originating from mesoderm, UCMSC-EVs enhance heme oxygenase-1 activity in mice with acute lung injury to inhibit apoptosis. This reduction in cell death is achieved by inducing the expression of detoxification enzymes and antioxidant proteins [133]. Similarly, miR-30b-3p delivered *via* BMMSC-EVs has been demonstrated to inhibit cell apoptosis by downregulating serum amyloid A3 [134]. Kaur et al. demonstrated that BMMSC-EVs attenuate experimental autoimmune uveoretinitis progression in mice by triggering apoptosis in retinal antigen-reactive T cells [135]. Wu et al. demonstrated that miR-100-5p-enriched AMSC-EVs function by inhibiting mTOR signaling in osteoarthritic mice [136]. Furthermore, IFN-β, secreted by UCMSCs, has also been proven to induce caspase-mediated apoptosis of cancer cells through JAK/STAT signaling pathway [137]. Subsequently, the release of ‘find-me’ signals by these apoptotic cells triggers the recruitment of phagocytes, facilitating the rapid clearance of the dying cells [138]. Stem cells originating from both the neural crest ectoderm and mesoderm have the ability to modulate cell apoptosis of both somatic cells and immune cells, thereby exerting therapeutic effects (Fig. 3).

### The angiogenic modulation effects of stem cells

Angiogenic modulation potential is a key characteristic of stem cells, typically referring to their ability to either promote or inhibit the development of new blood vessels in damaged tissue. Our objective is not to compare or rank the efficiency of different MSC types in these processes. Rather, we emphasize that MSCs across various tissue sources retain these regulatory capacities, which may be mediated by distinct underlying mechanisms.

Among stem cells from the neural crest ectoderm, small molecules and growth factors can induce SCAPs to adopt an endothelial-like state and form vessel-like tubular structures [139]. Furthermore, SHED-apoVs activate endothelial angiogenesis *via* the transcription factor EB-autophagy pathway [140], while SCAP-EVs deliver Cdc42 to upregulate endothelial CD31 expression, driving both cell migration and neovascularization [141].

Stem cells originating from the mesoderm exhibit a similar capacity to enhance vascularization. BMMSCs can be induced to differentiate into an endothelial phenotype, thereby increasing vascular density and improving cardiac function during chronic ischemic conditions [142], while explant-cultured UCMSCs undergo endothelial differentiation to restore perfusion in ischemic hindlimb models [143]. Paracrine signaling further drives this process; for example, AMSC conditioned medium contains high levels of bFGF and vascular endothelial growth factor (VEGF) capable of remodeling vascular networks [144]. Broadly, the therapeutic angiogenic potential of MSCs relies on this dual mechanism of vascular lineage differentiation and secretome-mediated paracrine signaling [145].

While BMMSCs promote vascular regeneration *via* bFGF and VEGF in rat myocardial infarction models [146], they attenuate corneal neovascularization by down-regulating VEGF following alkali burns [147]. Furthermore, MSC-mediated corneal reconstruction relies on anti-angiogenic and unique immunomodulatory mechanisms [148], with corneal-derived MSCs actively secreting anti-angiogenic factors such as soluble fms-like tyrosine kinase-1 (sFLT-1) and pigment epithelium-derived factor (PEDF) [149]. Therefore, the role of stem cells in angiogenesis, whether they promote or inhibit blood vessel formation, is contingent upon the microenvironment into which they are transplanted (Fig. 2).

In addition, stem cells from different embryonic lineages exhibit context-dependent angiogenic modulation. Depending on the tissue microenvironment, stem cells may promote angiogenesis through endothelial-like differentiation and the release of pro-angiogenic factors, or inhibit pathological neovascularization by secreting anti-angiogenic mediators, particularly in avascular tissues such as the cornea.

Moreover, stem cells from both embryonic lineages modulate apoptosis of somatic and immune cells. Stem cells originating from the neural crest ectoderm and their EVs inhibit excessive apoptosis by regulating intrinsic and extrinsic apoptotic pathways, whereas stem cells originating from the mesoderm and their EVs can suppress apoptosis in injured tissues or induce apoptosis in pathogenic immune cells and cancer cells through signaling pathways associated with caspases, Bcl-2 family proteins, and cytokine-mediated signaling.

## The interplay of germ layer origin, epigenetic memory, and niche in controlling stem cell fate

MSCs exhibit differentiation capacities and secretome profiles that are governed by their embryonic origin, epigenetic landscape, and niche-specific cues [150].

### Germ layer origin

The biological function of stem cells is governed by their germ layer origin, which restricts cross-lineage differentiation in vivo. Circa 2000, early frequently employed X/Y-chromosome fluorescence in situ hybridization (FISH) [151]-based tracking suggested MSCs could easily breach germ layer boundaries *via* transdifferentiation, sparking initial optimism [152–154]. However, rigorous Cre/loxP lineage tracing by Alvarez-Dolado et al. (2003) revealed these trans-germ-layer events were predominantly experimental artifacts, specifically in vivo cell fusion [155]. Subsequent studies confirmed that true transdifferentiation is rare, with artifacts often stemming from cell fusion [155, 156] or residual pluripotent cells [157]. While in vitro exogenous cues can force multi-germ-layer induction [23, 158–161], cellular identity remains strictly maintained in vivo in non-pathological contexts [162]. This persistent difficulty in crossing developmental boundaries underscores the inherently limited nature of MSC plasticity—defined as developmental potential transcending the differentiated lineages or germ layers of their tissue of origin [163, 164]. Stringent modern fate-mapping, including DNA-based molecular barcoding technology [165] and genome editing of synthetic target arrays for lineage tracing (GESTALT) [166], has firmly established this restricted plasticity. Consequently, understanding the robust constraints of germ layer origin has refined the evaluation of MSC potency, accompanied by a steadily growing body of research investigating paracrine-mediated repair mechanisms [167, 168]. Accumulating evidence supports the therapeutic potential of intra-germ-layer stem cell differentiation for addressing tissue defects. Employing an ex vivo heterotypic culture, Bejar et al. demonstrated that embryonic dermal signals could induce adult esophageal epithelium to transdifferentiate into skin lineages of the same germ layer. Although highly inefficient, this intra-germ-layer plasticity successfully yielded hair follicle-like structures [169]. Utilizing dual genetic lineage tracing, Pu et al. identified a biliary-derived transitional liver progenitor cell (TLPC) exhibiting restricted bipotency. Mechanistically, Notch signaling governs initial TLPC formation, while Wnt/β-catenin drives its transdifferentiation into hepatocytes rather than reversion to the biliary lineage, elucidating the pathways of transdifferentiation-assisted liver regeneration [170]. Furthermore, while previous studies demonstrated that BMMSCs could generate neural cell types alongside mesodermal tissues, this neurogenic capacity is driven by a distinct cell subtype of truncal neural crest origin within the long bone marrow, rather than the coexisting population of mesodermal origin [171]. Collectively, these findings indicate that while robust evidence for in vivo cross-germ-layer transdifferentiation remains elusive, intra-germ-layer plasticity provides a promising avenue for clinical intervention. In scenarios where lineage-specific stem cells are inaccessible, stem cells originating from the same germ layer may serve as viable alternatives. For instance, in the reconstruction of maxillofacial defects, DMSCs of neural crest origin—sharing the same germ layer with the jaw bone—offer a practical and accessible therapeutic substrate [13, 26].

The limited plasticity observed in MSCs is likely governed by their intrinsic genetic architecture. A subgroup of homeobox genes encoding transcriptional regulators, *HOX* genes serve as master determinants of both cell identity/fate and lifelong positional memory, thereby establishing the regional specialization observed in mature tissues [172, 173]. For instance, Yoshioka et al. established that *Hoxa10* expression distinguishes somite from cranial mesoderm derivatives in mice; notably, this *Hox-A* expression profile and localized hypermethylation are stably perpetuated in adult somite-derived muscle and satellite cells, recapitulating their embryonic origins [174]. Leucht et al. established that *HOX* expression profiles are linked to embryonic origin, with neural crest-derived mandibular niches being HOX-negative while mesoderm-derived tibial environments are HOX-positive [175]. Consequently, engrafting HOX-positive mesodermal MSCs into HOX-negative mandibular defects precipitates aberrant chondrogenesis and regenerative failure, driven by a critical transcriptional mismatch between donor cells and the host microenvironment [175, 176]. Wang et al. also have supposed that matching the positional identity of transplanted stem cells with that of the host environment, as reflected by their respective *HOX* profiles, is likely to be required to achieve regenerative healing [173]. Furthermore, comparative studies highlight germ layer-dependent biases: upon subcutaneous transplantation, DPSCs recapitulate their neural crest origin by generating odontoblast-like cells and a dentin-like matrix. Conversely, mesoderm-derived BMMSCs yield osteoblast-like cells and bone-like condensations under identical stimuli [177]. These indicate that stem cells in heterotopic sites still preferentially execute the repair program linked to their germ layer origin, rather than responding solely to the new environment. However, analysis of *HOX* gene expression across developmental lineages reveals that neural crest derivatives retain the positional signatures of their site of origin while concurrently acquiring the *HOX* profiles characteristic of their target destinations [178]. This plasticity stems from bivalent chromatin poising (H3K27me3/H3K4me2) at *HOX* clusters [179]. Cues such as retinoic acid or WNT signaling drive HOX-positive identity shifts through H3K27me3 depletion [180, 181], explaining why DMSCs also exhibit favorable outcomes when repairing mesoderm-derived tissues, reflecting their remarkable adaptability and regenerative capacity [182].

### Epigenetic memory

Among the central epigenetic mechanisms controlling gene expression in MSCs are DNA methylation, histone modifications, and chromatin remodeling [183]. Traditional models depict differentiation as a unidirectional cascade triggered by extrinsic cues. Recent research has revealed a more complex regulatory architecture. Epigenetic memory is encoded through a dynamic, reversible epigenetic cycle that is coordinated with the cell cycle machinery [184]. This multiphasic system enables proliferating stem cells to both maintain a stable gene expression program and, during transient periods of weakened epigenetic signaling, switch to differentiation in response to developmental cues [184].

Menezes et al. have shown that pluripotent stem cell responses to differentiation cues are predominantly preset by the epigenome. Specifically, epiblast DNA methylation primes WNT-driven germ layer commitment, emphasizing that it is lineage-specific methylation at gene loci that predetermines cell fate [185]. This developmental trait is also prevalent in MSCs. Although phenotypically undifferentiated, MSCs undergoing determination experience a profound molecular transition. This step entails the selective demethylation of promoters associated with key fate-determining factors, coupled with the simultaneous di-methylation of promoter regions belonging to late-differentiation lineages to ensure proper developmental timing [186]. Ultimately, this epigenetic priming provides the developmental competence necessary to translate extrinsic signals into site-specific programs [187]. This evidence indicates that under natural conditions, faced with identical differentiation cues, it is the gene-orchestrated epigenetic landscape—not the extrinsic signals—that specifies lineage differentiation bias.

Epigenetic memories acquired by stem cells remain dormant during homeostasis, yet upon a secondary challenge they are unleashed to confer broadened regenerative capacity—a trained-immunity-like phenomenon that can be either maladaptive or beneficial. Notably, such memories can endure for almost 2 months in mice (equivalent to 5-6 human years), highlighting a therapeutically exploitable window [188]. This provides the mechanistic basis for disease microenvironment preconditioning (DMP), a strategy wherein MSCs are subjected to diverse biochemical and biophysical stimuli in vitro—including 3D scaffolding, mechanical forces, and hypoxia—to program a favorable functional state [189]. Additionally, the therapeutic efficacy of stem cells depends upon their genetic and epigenetic stability, as these factors govern the robust paracrine output necessary for successful tissue regeneration [190].

### Niche

The stem cell niche is an anatomically discrete, specialized microenvironment that maintains direct contact with resident stem cells. Comprising heterogeneous supporting cell populations, this niche provides the extrinsic cues necessary to orchestrate the balance between self-renewal and lineage commitment, thereby ensuring developmental fidelity and long-term tissue homeostasis [191]. The microenvironment, through epigenetic regulation, cooperates with the germ layer origin to dictate stem cell differentiation and paracrine activity [150], with a particularly pronounced influence on the latter [192].

While in vitro growth factor supplementation effectively drives targeted stem cell specialization, the pleiotropic and often antagonistic signaling within the in vivo milieu frequently leads to a deviation from the anticipated outcome. For instance, BMMSCs exhibit robust in vitro chondrogenic capacity when cultured in 3D or supplemented with TGF-β isoforms [193, 194]; however, in vivo ectopic transplantation reveals a propensity for endochondral ossification, which hinders the establishment of a stable chondrogenic identity [195]. Even in vivo, the distinct signaling milieus of spatially disparate anatomical niches exert differential regulatory control over a uniform cell population, driving the specification of divergent subtypes. Han et al. demonstrated that the local cartilage microenvironment decisively dictates the fate of AMSCs originating from the mesoderm [196]. Specifically, within articular and meniscal niches—both of mesodermal origin—AMSCs undergo hyaline- and fibrocartilage-like differentiation, respectively [196]. This underscores the essential stem cell–niche interplay required for subtype-specific regeneration. Notably, while these intra-germ-layer transitions are well-supported, current lineage-tracing studies have yet to provide evidence of AMSC differentiation into non-mesodermal lineages. In MSC transplantation, the primary regenerative mechanism is increasingly attributed to the secretome rather than direct differentiation [197], as evidenced by the transient and limited nature of in vivo engraftment [192]. This shift toward paracrine-mediated signaling offers a more compelling and innovative therapeutic paradigm [198]. Hypoxic preconditioning recapitulates the physiological low-oxygen niche, priming MSCs for enhanced chondrogenic differentiation and augmenting their paracrine secretome composed of soluble factors and EVs [199, 200]. Notably, the resultant therapeutic efficacy is driven not by a quantitative increase in EV biogenesis, but by a qualitative reprogramming of EV cargo toward an anti-fibrotic miRNA signature [201]. Low-dose IFN-γ epigenetically primes MSCs toward an immunomodulatory phenotype by selectively remodeling the enhancer landscape [202]. EVs from UCMSCs primed with IFN-γ and TNF-α exhibit enhanced immunosuppressive potential by upregulating anti-inflammatory miRNAs (miR-139-5p, miR-140-5p, and miR-214-5p) [203]. Elucidating the mechanisms of microenvironmental stem cell regulation provides a robust framework for clinical translation. DMP—designed to enhance physiological resilience within the in vivo niche or augment paracrine potency—may be leveraged to optimize functional tissue regeneration [189, 204, 205].

## Recent advances in stem cell therapy in preclinical and clinical trials

### Applications of stem cells originating from the surface ectoderm

For stem cells originating from the surface ectoderm, LESCs possess the ability to treat corneal diseases. Oie et al. performed a clinical trial utilizing autologous cultivated LESCs to treat unilateral limbal stem cell deficiency (LSCD) and facilitated corneal epithelial reconstruction in patients [206]. However, in allotransplantation, the limited availability of donor eyes and the necessity for prolonged immunosuppressant therapy to reduce the risk of graft rejection still remain issues that need to be addressed, while in autotransplantation, especially for unilateral LSCD, there is a risk of damaging the healthy cornea when obtaining limbal tissue [207]. The transplantation of LESCs has achieved great advances in corneal diseases, but there are still limitations to their clinical use due to their availability.

### Applications of stem cells originating from the neural crest ectoderm

MSCs originating from the neural crest ectoderm show great potential in tissue reconstruction, such as nerve tissue and craniofacial bone tissue. In nerve tissue repair, MSCs are predominantly employed in rodent experimentation [208–210]. For example, Pereira et al. reported that SHEDs could enhance mandibular branch regeneration in the rat facial nerve [208]. Furthermore, prevascularized DPSC-embedded constructs have the potential to enhance and regulate spinal cord injury repair in rats [210]. In craniofacial bone repair, numerous clinical trials have been conducted to address alveolar bone loss resulting from periodontitis, with significant therapeutic efficacy reported. Ferrarotti et al. demonstrated that the application of autologous DPSCs in periodontitis patients led to a significant improvement in clinical periodontal regeneration parameters one year post-treatment [211]. Sánchez et al. also confirmed the safety of utilizing autologous PDLSCs for the treatment of periodontitis in patients [212]. However, the investigation of cranial bone defect treatments is still in the preclinical phase [213], with research conducted on animal models, such as swine [214]. Further clinical studies are required in order to elucidate their therapeutic potential. The capacity of multidirectional differentiation and immunoregulation also endows these MSCs with therapeutic potency in tissues from the endoderm such as the liver, intestine, bladder, and pancreas. In mice with liver fibrosis, transplanted SHEDs improve hepatic dysfunction through directly transforming into hepatocytes [215] and inducing proliferation of stem cells and native hepatocytes [216]. For intestinal diseases, DPSCs, transduced with the HGF gene, alleviate ulcerative colitis in rats through transdifferentiating into intestinal stem cell-like cells [217]. Notably, the majority of research progress remains confined to preclinical models, whereas the treatment of diabetes has been subjected to clinical trials. In patients with type 2 diabetes mellitus, a clinical trial demonstrated that the intravenous infusions of SHEDs improved glucose metabolism and islet function [218]. In general, MSCs originating from the neural crest ectoderm can treat diseases from various germ layers and have been extensively applied in preclinical and clinical trials for their safety and availability. Consequently, expanding literature underscores the therapeutic superiority of neural crest-originated MSCs in neural and craniofacial regeneration [219]. The clinical translation of neural crest-originated MSCs is raising despite their sourcing constraints and translational inertia [219, 220].

### Applications of stem cells originating from the mesoderm

**Fig. 4:**
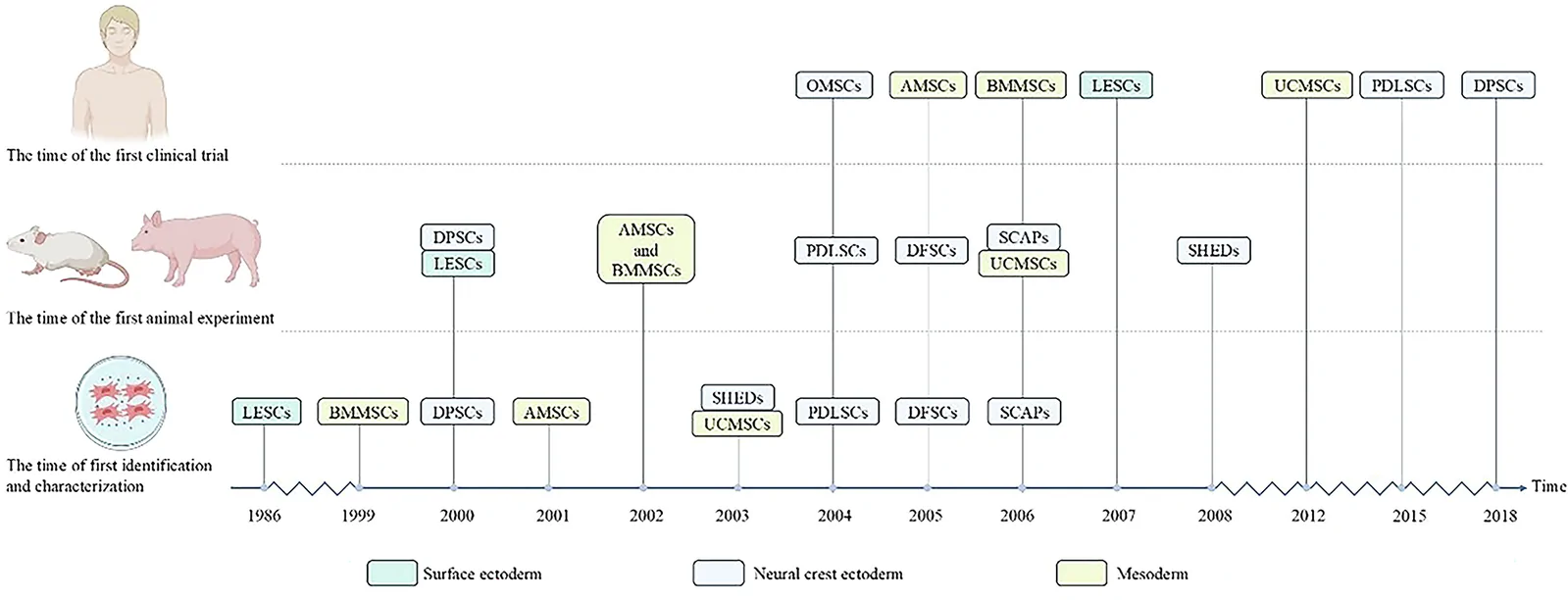
Comparison of clinical applications of stem cells originated from the three germ layers. This figure compares the timeline of the first identification and characterization, animal experiments, and clinical trials of stem cells originating from different germ layers. The green section represents LESCs originating from the surface ectoderm, the blue section represents DMSCs from the neural crest ectoderm, and the yellow section represents BMMSCs, UCMSCs, and AMSCs originating from the mesoderm.

**Fig. 5:**
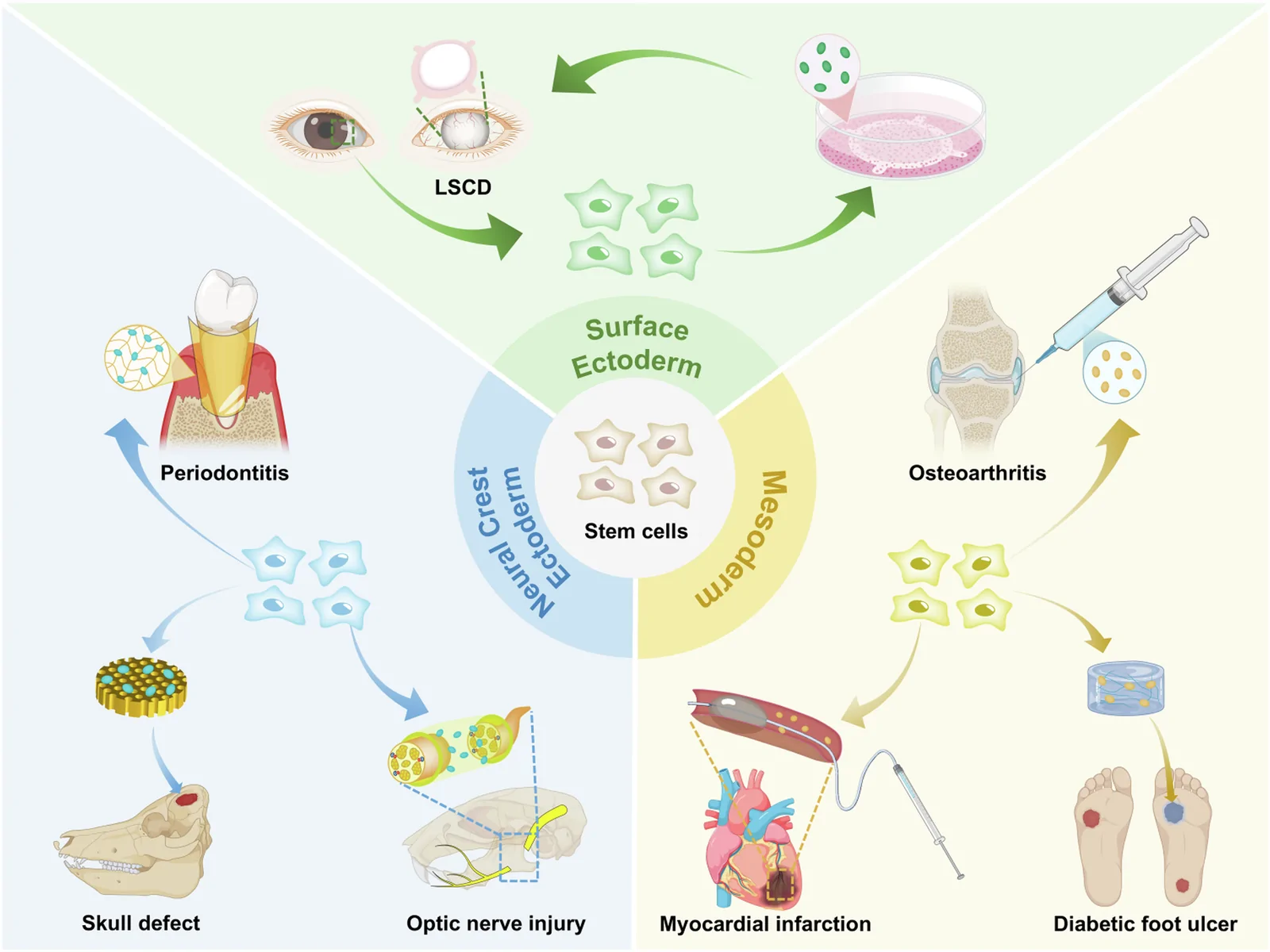
Applications of stem cells from different germ layers in corresponding germ layer-associated diseases. Stem cell therapies tailored to germ layer origin have shown intrinsic, layer-specific efficacy in translational studies. Limbal epithelial stem cells (LESCs) originating from the surface ectoderm restore the corneal surface in limbal stem cell deficiency (LSCD). Periodontal ligament stem cells (PDLSCs) originating from the neural crest ectoderm slow chronic periodontitis progression and support craniofacial bone regeneration. Bone marrow mesenchymal stem cells (BMMSCs) originating from the mesoderm regulate osteochondral remodeling to mitigate osteoarthritis pathogenesis.

The proliferation of clinical applications for stem cells originating from the mesoderm, such as BMMSCs, AMSCs, and UCMSCs, could be partially credited to the more extensive research history that these cells have been subjected to (Fig. 4) [53, 55, 56, 58, 211,221–236]. To date, over 1100 clinical trials have been registered on www.ClinicalTrials.gov concerning these cells. For neural diseases, Mohseni et al. indicated that intrathecal administration of AMSCs was safe and showed promising efficacy in patients with spinal muscular atrophy [237]. As for skin wound healing, Mrozikiewicz-Rakowska et al. reported that topical application of AMSCs resulted in significant improvement in patients with diabetic foot ulcer treatment [238]. These cells also have great potential in knee osteoarthritis. A phase 3 clinical trial demonstrated that intra-articular injection of autologous AMSCs elicited significant analgesic and functional benefits in patients with osteoarthritis [239]. Furthermore, Intra-articular administration of autologous BMMSCs [240] or UCMSCs secretome [241] has also been shown to present a promising therapeutic approach for patients with osteoarthritis. However, a meta-analysis of existing clinical trials on osteoarthritis treatment with MSCs indicated that AMSCs exhibited greater stability in their therapeutic outcomes compared to BMMSCs [86]. For cardiac diseases, Zhang et al. reported that there was insufficient evidence to prove the therapeutic efficacy of intracoronary autologous BMMSCs transplantation in patients with ST-segment elevation myocardial infarction [242]. Moreover, the immunoregulation property also endows stem cells originating from the mesoderm with potency of treating autoimmune diseases. For example, Petrou et al. implanted autologous BMMSCs intrathecally or intravenously and certified the clinical efficacy in patients with active progressive multiple sclerosis [243]. The therapy of UCMSCs and disease-modifying anti-rheumatic drugs can be a safe and effective therapeutic option for rheumatoid arthritis patients [244]. Taken together, all these preclinical and clinical trials establish the recognition of stem cells’ therapeutic effects in various diseases, although additional research is still required to fully elucidate their potential and to optimize clinical applications (Table 2, Fig. 5).

**Table 2:** Application and research progress of stem cells in various diseases.

Germ layer	Cell	Application	Research progress	References
Surface ectoderm	LESCs	LSCD	Clinical Trial: Phase 2	[183]
Neural crest ectoderm	SHEDs	Parkinson’s disease	Animal experiments: rats	[154]
		Hepatic dysfunction	Animal experiments: mice	[161]
		Type 2 diabetes mellitus	Clinical Trial: Early Phase 1	[218]
	DPSCs	Spinal cord injury	Animal experiments: rats	[210]
		Craniofacial bone defects	Animal experiments: swine	[214]
		Ulcerative colitis	Animal experiments: rats	[217]
	PDLSCs	Optic nerve injury	Animal experiments: rats	[281]
		Periodontitis	Clinical Trial: Phase 2	[212]
	SCAPs	Spinal cord injury	Animal experiments: rats	[282, 283]
Mesoderm	BMMSCs	Osteoarthritis	Clinical Trial: Phase 2	[240]
		Myocardial infarction	Clinical Trial: Phase 3	[242]
		Acute-on-chronic liver failure	Clinical Trial: Phase 2	[284]
	AMSCs	Osteoarthritis	Clinical Trial: Phase 3	[239]
		Spinal muscular atrophy	Clinical Trial: Phase 1	[237]
		Epithelium or mucosa defects	Clinical Trial: Phase 2	[238]
	UCMSCs	Osteoarthritis	Clinical Trial: Phase 2	[241]
		Dental pulp regeneration	Clinical Trial: Phase 2	[285]

Mapping of stem cells originating from three embryonic germ layers (surface ectoderm, neural crest ectoderm, and mesoderm) to targeted clinical indications. The table outlines key disease targets, experimental models (preclinical small/large animal models or specific clinical trial phases), and representative supporting literature.

**Fig. 6:**
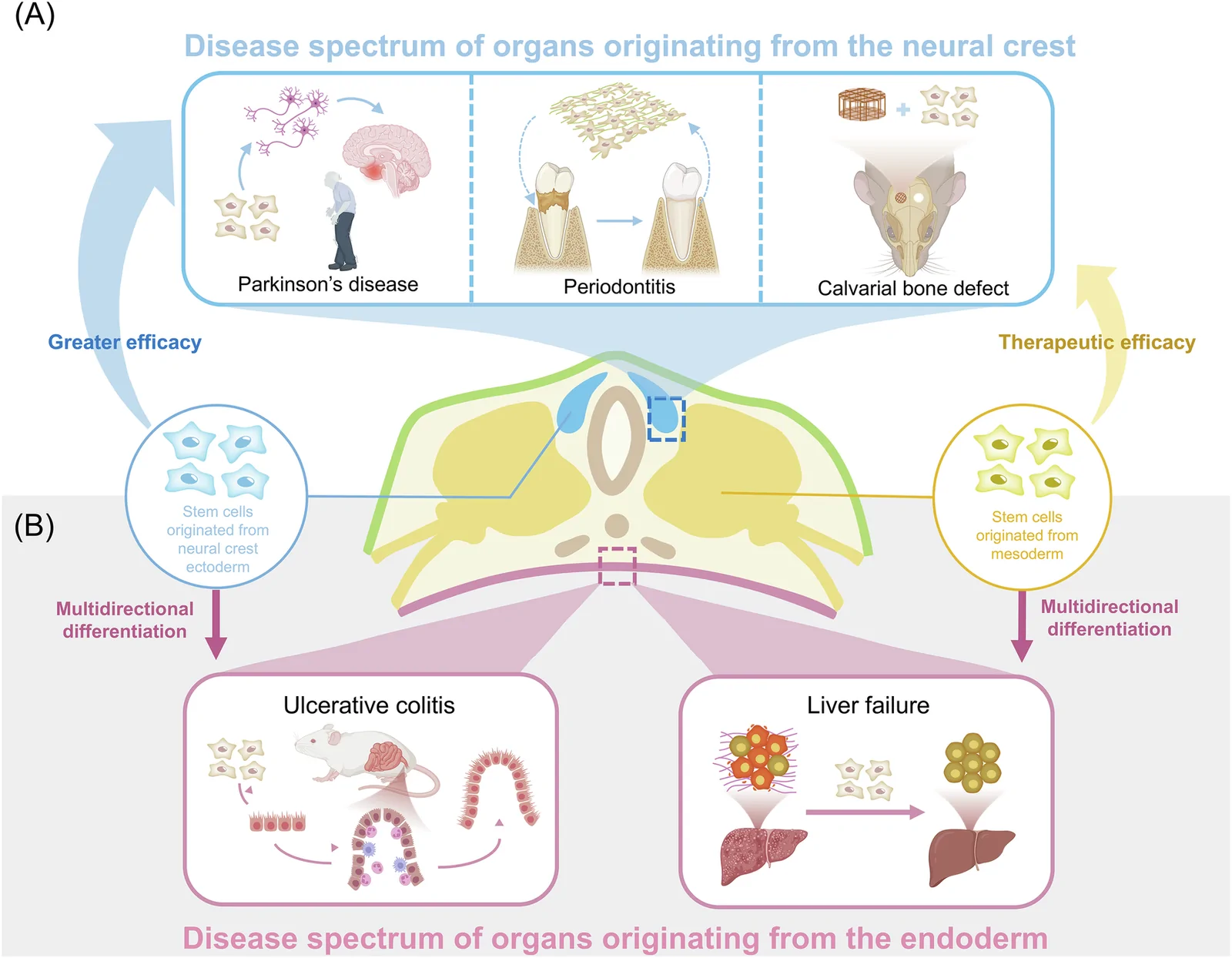
Stem cells originating from the neural crest ectoderm and mesoderm in germ layer-specific disease therapy. **A** Stem cells originating from the neural crest ectoderm outperform mesoderm-derived stem cells in treating neural crest ectoderm-derived disorders, including Parkinson’s disease, periodontitis, and craniofacial bone defects. **B** The inherent multipotency and immunomodulatory capacity of stem cells originating from either the neural crest ectoderm or the mesoderm enable their application in endoderm-derived organ disorders, such as ulcerative colitis and liver failure.

In view of the considerable diversity among stem cells, it is reasonable to inquire about the factors that influence the selection of specific stem cells for therapeutic purpose in clinical applications. Here, we propose that the germ layer origin may serve as an effective basis for our selection of corresponding stem cells when treating various tissue damage. In nerve tissue repair, secretions of DMSCs exhibited enhanced neuroprotective effects compared to those of MSCs originating from the mesoderm [27, 28]. Sharma et al. identified DPSCs as preferable to BMMSCs for Parkinson’s disease therapy due to their enhanced neural differentiation potential and neuron-regenerating capabilities [29]. Additionally, in a rat calvarial bone defect model, Srinivasan et al. demonstrated that MSCs originating from the neural crest had significantly higher cellularity and exhibited enhanced new bone formation compared to BMMSCs [13]. Furthermore, a recent study indicated that PDLSCs demonstrated enhanced osteogenic and periodontal potential compared to BMMSCs and AMSCs [30]; meanwhile, autologous PDLSC sheets were validated for safety and efficacy in patients with severe periodontitis [31]. In general, although MSCs originating from the neural crest ectoderm and MSCs originating from the mesoderm both possess the capacities for tissue repair, these results indicate that stem cells from the same germ layer origin may exhibit enhanced therapeutic efficacy (Fig. 6).

### Applications of induced pluripotent stem cells (iPSCs)

iPSCs, generated by reprogramming somatic cells with specific transcription factors, exhibit the hallmark characteristics of pluripotency [245, 246]. Owing to the facile procurement of donor somatic cells and the circumvention of major ethical hurdles, iPSCs provide a highly versatile platform, facilitating their extensive utilization [247].

Directing lineage commitment in iPSCs remains a major challenge for clinical translation [248]. Although somatic cells from all three germ layers are amenable to reprogramming [249–253], iPSCs preferentially differentiate into tissues of the same germ layer. For instance, studies reported variable morphogen responses among iPSC lines, demonstrating that iPSCs derived from brain-derived fetal neural stem cells generated more neurons and exhibited enhanced cortical and striatal engraftment compared to fibroblast-derived iPSCs, suggesting a more permissive neural niche for cells sharing a neural crest origin [253]. Similarly, Chlebanowski et al. found that mesodermal peripheral blood mononuclear cell-derived iPSCs exclusively form bone and muscle within teratomas [254]. This lineage bias is consistently observed across mesodermal and endodermal differentiation. Mesoderm-derived iPSCs exhibit enhanced mesodermal differentiation capacity relative to ectodermal or endodermal counterparts [255–257]. Specifically, BMMSC-derived iPSCs display enhanced cardiomyogenic potential compared to keratinocyte-derived iPSCs [256]. For endodermal lineages, utilizing primary hepatocyte-derived iPSCs bypasses the germ-layer transition, optimizing differentiation into liver-specific cell types [258]. Furthermore, human pancreatic islet β-cell-derived iPSCs preferentially differentiate toward endodermal lineages in vitro [259].

This germ-layer bias is primarily driven by genetic backgrounds and the incomplete erasure of epigenetic memory [253, 260]. For example, whole-genome and neural transcriptomic profiling segregated neural crest ectoderm-from mesoderm-derived iPSC-neural progenitor cells, revealing gene expression patterns shared between fetal neural stem cells and neural crest ectoderm but absent in mesodermal derivatives [253]. Accumulating evidence confirms that retained lineage-specific epigenetic signatures, such as histone modifications and DNA methylation, direct donor-lineage differentiation preferences [257]. Specifically, Kim et al. demonstrated that heart endocardium-derived circulating multipotent stem (CiMS) cell-iPSCs differentiate more readily into mesoderm than dermal fibroblast-iPSCs, a phenomenon mediated by markedly elevated H3K4me3 levels at the Brachyury T promoter region [261]. In summary, iPSCs retain intrinsic differentiation biases dictated by germ layer-specific epigenetic memory, rendering the original somatic lineage a critical determinant of differentiation propensity and potential.

During embryogenesis, pluripotent stem cells (PSCs) develop heterogeneity and transition from a naive to a primed state, committing to specific fates *via* spatiotemporally regulated intrinsic and extrinsic factors [262]. This commitment is governed by lineage-specific transcription factors (specifiers) that dictate cell identities [263] by accessing chromatin to selectively activate or silence target genes [264]. Key examples include ETV2 for endothelial specification [265], FOXA1 for hepatic commitment [266], and ASCL1 for neurogenesis [267]. These intrinsic programs require extrinsic triggers; during hematopoiesis, for instance, VEGF receptor binding activates MAPK, NOTCH, and HIPPO signaling to drive downstream transcriptional networks [268]. To recapitulate development in vitro, various strategies are employed to induce these specifiers in iPSCs. These include microenvironmental modulation (e.g., TGF-β for mesenchymal induction) [269, 270], transcription factor overexpression (e.g., NGN2 for excitatory neurons) [271, 272], epigenetic regulation (e.g., KDM4A-dependent histone demethylation for pancreatic β-cells) [273], and genetic reprogramming (e.g., CRISPR-mediated safe-harbor delivery of ISL1 and LHX3 for spinal motor neurons) [274]. Physical developmental niches are similarly mimicked *via* tuned substrate stiffness [275] or 3D organoid cultures [276]. Addressing current variations in differentiation efficiency, donor heterogeneity, and epigenetic memory may represent a vital opportunity to fully realize the clinical potential of iPSC translation [253].

## Conclusion

The application of MSCs in the treatment of injured tissues has constituted a prominent area of investigation within the scientific community. Previous studies have predominantly focused on the use of stem cells sourced from the same tissue or organ for treating the corresponding disease. Recently, there have been numerous publications comparing the gene expression and function of stem cells originating from disparate germ layers, such as DMSCs from the neural crest ectoderm and BMMSCs from the mesoderm. However, there is a lack of comprehensive literature that integrates the biological properties and clinical applications of stem cells from each germ layer.

This article aims to explore the classification of stem cells based on their germ layer origin and the use of this classification in clinical applications to improve therapeutic outcomes. Firstly, there is literature indicating that although stem cells can differentiate into various lineages, they tend to differentiate more readily into their lineage of origin [277]. Furthermore, a study was carried out to compare the gene expression profiles of PDLSCs originating from the neural crest ectoderm and UCMSCs originating from the mesoderm [278]. The results revealed that the Homeobox genes, including *HOX*, exhibited the most significant differential expression patterns [26]. Intriguingly, Leucht et al. reported that mandibular microenvironment and stem cells originating from the neural crest ectoderm were HOX-negative, while tibial microenvironment and stem cells originating from the mesoderm adopted a HOX-positive profile. Thus, stem cells originating from the mesoderm were proved to differentiate into chondrocytes following transplantation into mandibular defects derived from the neural crest ectoderm, whereas differentiate into osteoblasts in tibial defects derived from the mesoderm [175]. This appears to indicate that, at the genetic level, stem cell therapy may exhibit enhanced efficacy when applied to tissues derived from the same germ layer.

Undeniably, factors beyond genetics, such as epigenetic regulation and niche, modulate stem cell functional properties and clinical therapeutic outcomes. Although the relative weight of each variable remains to be elucidated, we contend that developmental—specifically germ layer—origin plays a pivotal and non-negligible role in governing stem cell behavior. Furthermore, we also provide a comprehensive overview of iPSCs, which have become a major focus of extensive contemporary research. In summary, this review discusses stem cells originating from various germ layers and summarizes their therapeutic mechanisms and clinical applications, providing a strong basis for selecting stem cells with better efficacy in treating corresponding diseases in clinical practice.
